# Commentary: The potential of systemic immune-inflammation index in predicting outcomes of facial palsy in patients with Ramsay Hunt syndrome treated by acupuncture

**DOI:** 10.3389/fneur.2025.1734415

**Published:** 2025-12-15

**Authors:** Jia Chen, Lihua Xuan

**Affiliations:** 1 The First School of Clinical Medicine, Zhejiang Chinese Medical University, Hangzhou, Zhejiang, China; 2 Department of Acupuncture and Moxibustion, The First Affiliated Hospital of Zhejiang Chinese Medical University, Zhejiang Provincial Hospital of Chinese Medicine, Hangzhou, Zhejiang, China

**Keywords:** Ramsay Hunt syndrome, facial palsy, acupuncture, systemic immune-inflammation index, prognosis, biomarker, neuroinflammation, viral neuropathy

## Introduction

We read with great interest the recent article by Fang et al. (1), which explored the role of the systemic immune-inflammation index (SII) as a predictor of acupuncture efficacy in patients with Ramsay Hunt syndrome (RHS)-related facial palsy. The authors should be commended for their pioneering investigation into a novel, readily accessible biomarker at the intersection of neuroinflammation, viral neuropathy, and complementary medicine. Their findings suggest that SII may serve as a useful tool for stratifying patients and personalizing treatment strategies—a promising step toward more objective assessment in acupuncture therapy for neurological disorders. We would like to engage in a constructive discussion regarding several methodological and interpretative aspects. The following commentary aims to elaborate on these points to help refine the conclusions and guide future research in this promising field.

## Heterogeneity in acupuncture stimulation

Although the authors describe a “standardized acupuncture protocol,” the manual stimulation applied differed based on the time from symptom onset (mild stimulation within 1 week, stronger stimulation thereafter). This introduces a potential source of heterogeneity in treatment delivery. Variation in needle stimulation intensity has been shown to influence neuroimmune responses and clinical outcomes in facial palsy (2). For instance, stronger manual stimulation may activate distinct neural pathways, such as A-delta fibers and the descending pain modulatory system, which could differentially modulate local and systemic inflammation (3). Without quantifying or randomizing stimulation parameters, it remains challenging to ascertain whether the observed association between SII and outcomes is independent of this procedural variability. Future studies should consider employing electroacupuncture with standardized parameters (e.g., fixed frequency and intensity) to enhance reproducibility and mechanistic interpretation.

## SII dynamics in the context of concomitant pharmacotherapy

All patients in the study received antiviral and corticosteroid therapy, yet the potential interaction between these treatments and SII was not explored in depth. Corticosteroids are potent immunomodulators that can rapidly alter neutrophil and lymphocyte counts—the very components of SII (4). A recent study by Chen et al. (5) demonstrated that dexamethasone administration significantly reduces SII within 48 h in patients with acute inflammatory conditions. Therefore, the baseline SII measured in this study may not fully reflect the pre-treatment immune-inflammatory status but rather a partially modulated state. It would be valuable to analyze whether the time interval from corticosteroid initiation to SII measurement influenced the results, or to consider serial SII measurements during treatment to capture its dynamic nature as a biomarker.

## Overlooking the role of viral load and specific immune response

The study focused exclusively on host inflammatory markers without accounting for viral load or specific antiviral immune responses. In RHS, varicella-zoster virus (VZV) reactivation drives not only inflammation but also adaptive immune activation. Studies have shown that VZV-specific T-cell responses correlate with recovery in herpes zoster-related complications (6). A low lymphocyte count—a key component of SII—may reflect VZV-induced immunosuppression or delayed viral clearance, rather than being solely an indicator of systemic inflammation. Thus, SII might potentially serve as a surrogate for inadequate antiviral immunity. Incorporating VZV DNA quantification via PCR or functional assays of VZV-specific T-cell activity (e.g., interferon-gamma release assays) in future studies could help disentangle these distinct pathophysiological mechanisms.

## Statistical and conceptual refinement of the predictive model

The combination of SII and body mass index (BMI) in a predictive model yielded only a marginal increase in the area under the curve (AUC) (0.840 vs. 0.839). While the authors suggest this combination “may still offer additional prognostic insight,” this interpretation warrants caution. The minimal improvement, coupled with the loss of BMI's statistical significance in the multivariate analysis, suggests that BMI adds limited value beyond SII alone in predicting acupuncture response. Furthermore, BMI is a crude measure of adiposity and may not accurately reflect visceral fat mass or pro-inflammatory adipose tissue activity. Future predictive models might benefit from incorporating direct measures of adipose-driven inflammation, such as leptin or adiponectin levels, which are more closely linked to immune dysregulation (7).

## Mechanistic speculation and the need for direct evidence

The discussion posits that acupuncture modulates the cholinergic anti-inflammatory pathway and the hypothalamic-pituitary-adrenal (HPA) axis, thereby potentially reducing systemic inflammation. While this is a plausible and increasingly researched hypothesis, the present study design does not provide direct evidence for the engagement of these specific mechanisms in the context of RHS. For instance, no physiological measurements of vagal tone (e.g., heart rate variability), cortisol, or specific inflammatory cytokines (e.g., IL-6, TNF-α) were performed. Recent work by Liu et al. (8) elegantly showed that electroacupuncture upregulates vagal activity and reduces IL-6 in a mouse model of sterile inflammation via a specific neuroanatomical circuit. However, such pathways remain to be conclusively verified in human facial palsy models. We encourage the authors and other researchers in the field to acknowledge this as an area for future validation and to design subsequent studies that integrate multi-modal assessments of neuro-immune function.

## Discussion

The study by Fang et al. provides a valuable foundation for using SII as a prognostic biomarker in RHS patients undergoing acupuncture. However, several methodological aspects warrant refinement to strengthen the clinical applicability of these findings. The heterogeneity in acupuncture stimulation parameters represents a potential confounder, as varying needle manipulation may differentially engage neuro-immune pathways (2, 3). The interpretation of a single baseline SII is complicated by concomitant corticosteroid administration, which rapidly alters its composite cell counts (4, 5); dynamic SII tracking is thus recommended. The pathophysiological context would be enriched by integrating viral load and VZV-specific immune markers to clarify whether SII reflects viral activity or pure host inflammation (6). Finally, the proposed cholinergic anti-inflammatory mechanism remains speculative without direct neuro-immune measurements (8). Future studies should prioritize standardizing acupuncture delivery, employing serial biomarker assessments, and incorporating virological and neuro-immune correlates to validate this promising prognostic approach.
